# A novel plant type, leaf disease and severity identification framework using CNN and transformer with multi-label method

**DOI:** 10.1038/s41598-024-62452-x

**Published:** 2024-05-22

**Authors:** Bin Yang, Mingwei Li, Fei Li, Yongbo Wang, Qiaokang Liang, Ruiyuan Zhao, Caihong Li, Jianwu Wang

**Affiliations:** 1 College of Electrical and Information Engineering, Hunan University, Changsha, 410082 China; 2 Key Laboratory of Visual Perception and Artificial Intelligence of Hunan Province, Hunan University, Changsha, 410082 China; 3 Cotton Sciences Research Institute of Hunan, Changde, 415101 China; 4 Hunan Research Institute for Development, Hunan University, Changsha, 410082 China

**Keywords:** Classification and taxonomy, Image processing, Machine learning

## Abstract

The growth of plants is threatened by numerous diseases. Accurate and timely identification of these diseases is crucial to prevent disease spreading. Many deep learning-based methods have been proposed for identifying leaf diseases. However, these methods often combine plant, leaf disease, and severity into one category or treat them separately, resulting in a large number of categories or complex network structures. Given this, this paper proposes a novel leaf disease identification network (LDI-NET) using a multi-label method. It is quite special because it can identify plant type, leaf disease and severity simultaneously using a single straightforward branch model without increasing the number of categories and avoiding extra branches. It consists of three modules, i.e., a feature tokenizer module, a token encoder module and a multi-label decoder module. The LDI-NET works as follows: Firstly, the feature tokenizer module is designed to enhance the capability of extracting local and long-range global contextual features by leveraging the strengths of convolutional neural networks and transformers. Secondly, the token encoder module is utilized to obtain context-rich tokens that can establish relationships among the plant, leaf disease and severity. Thirdly, the multi-label decoder module combined with a residual structure is utilized to fuse shallow and deep contextual features for better utilization of different-level features. This allows the identification of plant type, leaf disease, and severity simultaneously. Experiments show that the proposed LDI-NET outperforms the prevalent methods using the publicly available AI challenger 2018 dataset.

## Introduction

Plant diseases pose a threat to the global food production^1^. Accurate and timely identification of plant diseases is crucial to prevent disease spread and ensure food security^2–4^. Therefore, plant disease identification has thus become one of the most urgent problems that needs to be solved in the agricultural intelligence community.

Usually, plant disease identification is typically done through manual observation, which is known to be laborious and time-consuming^5,6^. With the rapid development of computer vision, machine learning has become one of the most popular technologies for identifying plant diseases. This is due to its learning ability, which can help to improve the accuracy and reduce the need for manual intervention. For example, Rumpf et al. used a support vector machine network to identify healthy and diseased sugar beet leaves^7^. Munisami et al. utilized k-nearest neighbor classifiers to identify plants based on shape features and color histograms^8^. Qin et al. evaluated the performance of supervised classification algorithms, including the naive Bayesian algorithm and regression trees algorithm, using the alfalfa diseases dataset^9^. Overall, machine learning algorithms rely on expert knowledge of diseases to construct handcrafted features. This may result in low identification accuracy in the field^10^.

Recently, an increasing number of deep learning-based models have been proposed for identifying plant diseases^11,12^. These models can automatically extract features of plant diseases, replacing the need for manually crafted features^13–16^. Two widely used deep-learning models for plant disease identification are convolutional neural network (CNN) and transformer^17–19^. Most of these models take the form of plant-disease-severity combination or plant-disease combination based on a single-branch model. For example, Zhao et al. proposed a DTL-SE-ResNet50 model for the identification of vegetable-disease combinations, such as tomato powdery mildew^20^. Gao et al. designed a single-branch network that combined the dual efficient channel attention module with ResNet for plant-disease identification^21^. Yang et al. to diagnosed crop diseases by taking the form of crop-disease-severity combination^22^. However, some research has indicated that this form extracts a significant amount of information from irrelevant image backgrounds rather than useful information^14^. This also increases the number of categories due to the combination of plant, disease, and severity^23,24^. To enhance the ability to extract useful information and to avoid a large number of categories, some researchers use a novel multi-task network that uses a multi-branch architecture to separate plant, leaf disease, and severity, and identify them separately. For example, Yang et al. utilized a triple-branch swin transformer to separately identify diseases and severities^25^. Liang et al. proposed PD2SE-Net, a multi-task identification model based on Shuffle-Net-v2, capable of identifying diseases and their severities^26^. Wang et al. proposed a triple-branch trilinear-CNN model to achieve identifying crops and diseases^14^. Keceli et al. proposed a deep learning-based multi-task prediction system with two branches for classifying crops and diseases^27^. It has been found that this form reduces the number of categories while increasing the number of per category. However, this also leads to more model parameters and training complexities^28^. Therefore, it is also not the optimal.

When identifying plant diseases, it is important to provide information on the plant type, leaf disease, and severity. Such information could be used to select appropriate preventive measures. Although the aforementioned studies have considerably improved the accuracy, they often involve a large number of categories and complex network structures. Therefore, there is a need for a new method to identify plant, leaf disease and severity. This method should decrease the number of categories and increase the number of per category and avoid extra branches. Currently, we are unaware of any single-branch that can simultaneously identify plant, leaf disease and severity.

To tackle the aforementioned problems, this paper proposes a novel leaf disease identification network (LDI-NET) using a multi-label method. This method could combine the advantages of both single-branch and multi-branch models to simultaneously identify the plant, leaf disease and severity. In plant disease identification tasks, CNN and transformer are commonly used as the backbone network. CNN can capture local information, while transformer can capture long-range global information. Thereby, we designed a feature tokenizer module based on CNN and transformer to enhance the ability to extract features. The module expresses the multi-label information of plant diseases as the compact spatial feature tokens. The token encoder module facilitates information exchange among extracted tokens to obtain context-rich tokens. Meanwhile, we utilized a multi-label decoder model combined with a residual structure to fuse compact spatial feature tokens and context-rich tokens (fused token) for enhancing feature representation and identifying different labels simultaneously.

In summary, this paper focuses on three major objectives: (i) This paper proposes a novel model named LDI-NET, which utilizes the multi-label method for the simultaneous identification of plant type, leaf disease, and their respective severity. Unlike existing methods, LDI-NET uses a multi-label method to improve the accuracy of plant disease identification. (ii) A feature tokenizer module is designed to capture both local and long-range global information, which is crucial for accurate identification. This module facilitates the extraction of essential features from input images of the proposed LDI-NET model. Additionally, a token encoder module is utilized to facilitate information exchange. (iii) Our comprehensive experiments and evaluations conducted on the AI Challenger 2018 dataset demonstrate that LDI-NET achieves state-of-the-art performance in identifying plant diseases. These results highlight the effectiveness and superiority of LDI-NET compared to existing methods.

The subsequent sections of this paper are structured as follows: Section "Method" describes the LDI-NET in detail. Section "Experiments and results" presents the dataset, evaluation metrics and experiment results. Section "Discussion" shows the features visual analyses and discussions of ablation experiments. Finally, Section "Conclusion" summarizes this paper.

## Method

### Overview

**Figure 1:**
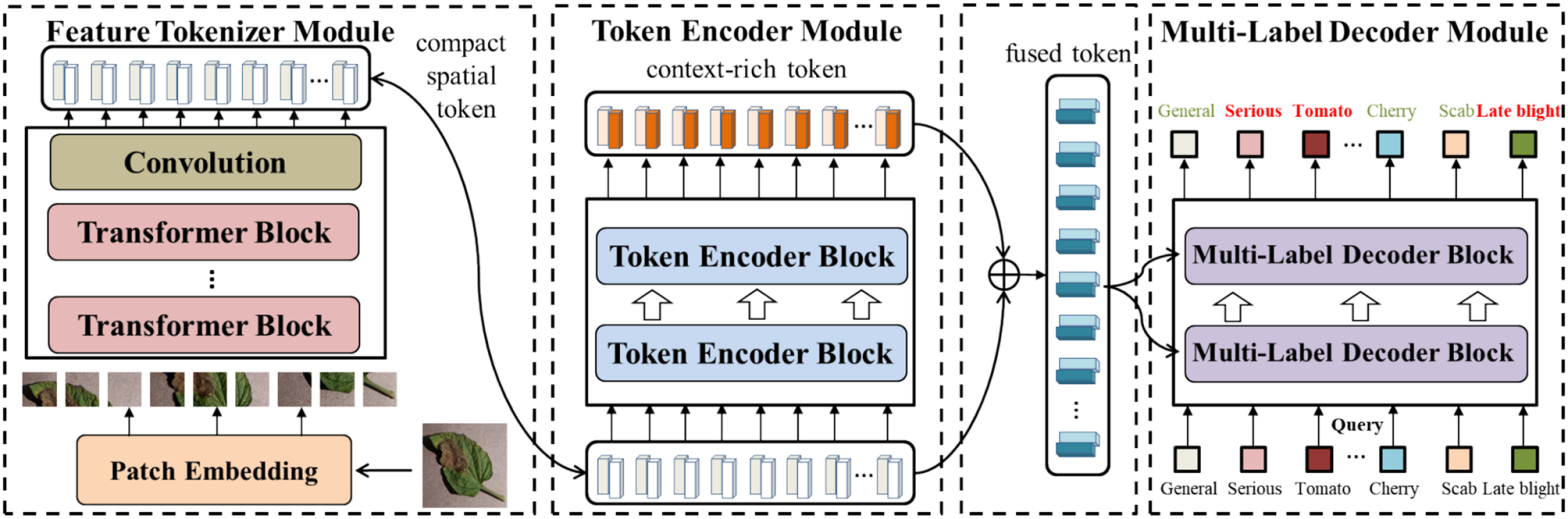
Architecture of the proposed LDI-NET.

Figure 1 shows the architecture of the LDI-NET, which comprises a feature tokenizer module, a token encoder module, and a multi-label decoder module. The feature tokenizer module is designed for extracting feature by combining the strengths of CNN and transformer. The token encoder module further establishes relationships among the features of plant type, leaf disease, and severity by facilitating information exchange. Moreover, the multi-label decoder module integrates fused tokens and adaptive feature embeddings to selectively extract features based on identifying plant, disease or severity, thus the accurate identification results for plant, leaf disease and severity can be obtained. The overall constitution of the LDI-NET is as follows:

1. The feature tokenizer module consists of four parts: Plant disease images are divided into patches. These patches are then flattened and fed into the transformer blocks to capture long-range global information. Next, a convolutional layer is connected after the transformer blocks to capture local information. In the end, the patches are flattened to compact spatial feature tokens, where each token corresponds to a specific aspect of the image such as color, texture or shape.
2. The token encoder module consists of two parts: Extracted tokens are fed into multi-head self-attention to facilitates information flowing that establish relationships among the features for plant, leaf disease and severity identification. Then the Multilayer Perceptron (MLP) structure further enhances the feature representation ability of model. As a result, the module generates tokens that are context-rich.
3. The multi-label decoder module consists of three parts: The self-attention takes adaptive feature embeddings as input for Q, K, and V. The cross-attention takes the outputs of the self-attention as input for Q, and the fused tokens as input for K and V. It selectively extracts leaf or disease region features based on plant, disease or severity identification. Extracted features are then fed into an MLP. The decoder learns the correlations between the labels and features to predict identification results.

### Feature tokenizer module

CNN^29^ and transformer^30^ are commonly utilized as backbone networks for feature extraction. While CNN can capture rich local information and provide inductive bias to improve the generalization ability of models^31,32^, it has limitations in capturing long-range global information due to the limited size of its receptive fields^33^. On the other hand, the transformer has shown great potential in capturing long-range global contextual visual information by utilizing the global relationship among spatial pixels^33,34^. However, the transformer blocks have a problem in capturing local information, and the lack of inductive bias leads to a low generalization ability of models^35^.

**Figure 2:**
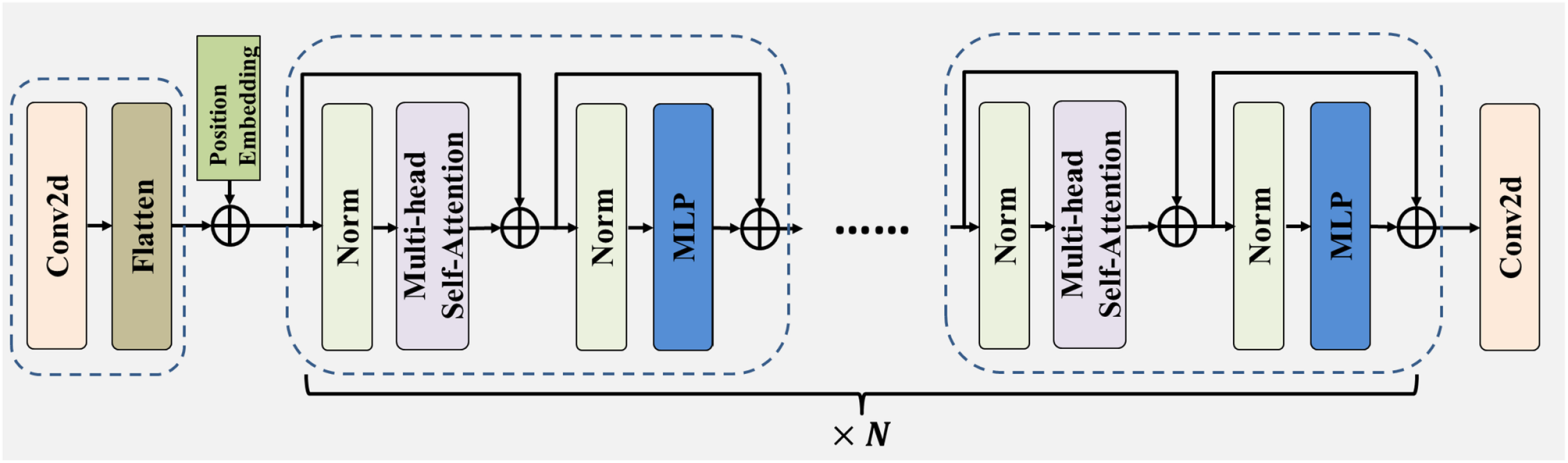
Architecture of the feature tokenizer module.

It can be found that CNN and transformer are complementary, thus there have been many studies devoted to combining CNN and transformer. For example, Zhang et al. designed a convolution transformer mixer that effectively combines the advantages of both for hyperspectral image classification^36^. Gu et al. used both CNN and transformer to segment remote sensing images^37^. Inspired by these works, this paper designs a hybrid module named feature tokenizer module. This module combines the strengths of CNN and transformer to improve the extraction ability of local and long-range global information of plant disease images. As illustrated in Fig. 2, the feature tokenizer module adopts a sliding convolution window of fixed size ($\text{convolution size}=16\times 16$ or $32\times 32$) to divide the image into patches (patch size=$16\times 16$ or $32\times 32$), which results in a disorderly arrangement of spatial position information. Therefore, position embeddings are added to each patch to preserve the original spatial position relationship. Then, the flattened patches are fed into the transformer blocks, which can extract long-range global features^38^. The transformer blocks lack the inductive bias and ability to capture local information. To address these issues, a convolutional layer is connected. This module can express the multi-label information among pixels as compact spatial feature tokens. The feature tokenizer module can be formulated as follows:

1 $$Y=\text{Conv}2\text{d}(\text{TB}\dots \text{TB}(\text{Conv}2\text{d}\left(\text{I}\right)))$$

where $Y$ denotes tokens, $\text{Conv}2\text{d}$ denotes convolution, $\text{TB}$ denotes the transformer block, and $\text{I}$ denotes plant disease image.

### Token encoder module

**Figure 3:**
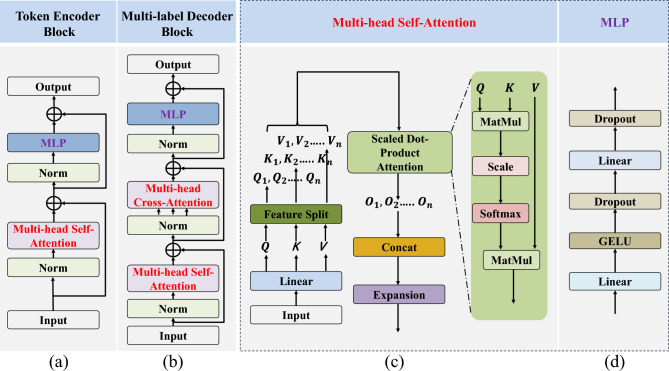
Architecture of the token encoder and the multi-label decoder block (**a**) architecture of the token encoder block, (**b**) architecture of the multi-label decoder block, (**c**) architecture of the multi-head self-attention, (**d**) architecture of MLP.

Most plant disease identification models use the extracted features directly for identification. This is a simple and straightforward method. However, this method is not the optimal for identifying multi-label plant diseases with complex labels, and it may decrease identification accuracy^26^. Studies have shown that the single-branch model discards the underlying cross-relatedness among the multi-branch model^39^. Therefore, the compact spatial feature tokens extracted by the single-branch feature tokenizer module cannot fully express the relationship between plant features, disease features and severity features. To overcome this limitation, the token encoder module is utilized to encode the input tokens by a matrix with hidden information^40^. It facilitates information exchange between tokens and mines the relationship between different features of plant diseases, resulting in an improved feature representation^41,42^. The token encoder module consists of two multi-label encoder blocks. Figure 3a illustrates the structure of the token encoder block, which consists of a multi-head self-attention (MSA) and an MLP. Additionally, necessary residual connections and norms are added^41^ to prevent model degradation and accelerate convergence during training.

(1) Multi-head Self-Attention (MSA): The structure of MSA is shown in the Fig. 3c, which can facilitate the exchange of multi-label information between tokens. The single-head self-attention (SA) selectively extracts multi-label features of plant diseases, without being limited by the size of the receptive field. The implementation of SA is based on trainable vector tuples (Query(Q), Key(K), Value(V)), which can filter out the multi-label information of the images. When the input sequence is ${X}_{Q}$, ${X}_{K}$ and ${X}_{V}$ which contain plant disease information, the values of Q, K and V can be obtained by projecting ${X}_{Q}$, ${X}_{K}$ and ${X}_{V}$ onto three matrices:

2 $$Q={X}_{Q}{W}^{Q}$$

3 $$K={X}_{K}{W}^{K}$$

4 $$V={X}_{V}{W}^{V}$$

where ${W}^{Q}$, ${W}^{K}$ and ${W}^{V}$ denote the learnable parameter matrices. When ${X}_{Q}$, ${X}_{K}$, and ${X}_{V}$ are equal, the SA can be formulated by calculating the similarity between $Q$ and $K$:

5 $$\text{SA}(\text{Q},\text{K},\text{V})=\text{softmax}(\frac{\text{Q}\cdot {K}^{T}}{\sqrt{{d}_{k}}})\cdot V$$

where ${K}^{T}$ represents the transpose matrix of $K$. The $\text{softmax}$ function performs a normalization operation that compresses the results of $\frac{\text{Q}\cdot {K}^{T}}{\sqrt{{d}_{k}}}$ to a range of 0–1 to obtain the attention matrix of different plant disease features^43^. Here, ${d}_{k}$ represents the scale of $\text{K}$. Dividing by $\sqrt{{d}_{k}}$ to counteract the gradient vanishing caused by $\text{softmax}$^44^. The occurrences of plant diseases are complex and there are many different forms of correlation between plant disease features, but the SA pays more attention to one type of feature information^34^. MSA can reduce the reliance on external information of the model and enhance the ability to capture internal correlations between features, thus it can promote information exchange between the plant, leaf disease and severity^41^. In order to enhance the adaptation to plant disease identification task, it is necessary to increase the number of SAs^44^. MSA can be introduced as follows:

6 $${\text{h}}_{i}=\text{SA}(\text{Q}\cdot {W}_{i}^{Q},K\cdot {W}_{i}^{K},V\cdot {W}_{i}^{v})$$

7 $$\text{MSA}(\text{q},\text{k},\text{v})=\text{Concat}({\text{h}}_{1},{\text{hd}}_{2}, \dots ,{\text{h}}_{\text{n}}){W}_{O}$$

where the index $i$ denotes the index of the $i\text{th}$ head in the MSA, and ${W}_{O}$ denotes a learnable mapping matrix. By utilizing MSA, which can allow the model to focus on different features of plant disease images, and different SAs are responsible for correlating these different features^45^. The use of MSA can integrate the information learned from different SAs and enhance the expression ability of the model.

(2) Multilayer Perceptron (MLP): As illustrated in the Fig. 3d, the MLP consists of linear layers, a GELU activation function and dropout layers. Studies have shown that networks based on the pure attention mechanism suffer from rank collapse during the training of models for identifying plant diseases. The MLP is introduced to increase its Lipschitz constant, thereby controlling the convergence speed and preventing the representation ability of self-attention networks from decaying exponentially with depth, leading to the degradation of the model^46^. Moreover, the MLP structure enhances the representation ability of the model by further transforming the extracted plant disease features using fully connected layers^47^.It improves the accuracy of multi-label identification for plant diseases.

After the compact spatial feature tokens of plant diseases are obtained by the feature tokenizer module, these tokens cannot establish sufficient connections among themselves. To address this issue, the token encoder module is employed to model among these tokens^48^. The tokens are fed into the MSA and the MLP. First. The MSA allows the free flow of information between tokens^45^, facilitating the exchange of information to focus on the interrelationships between plant, leaf disease and severity features^49^. It enhances the ability to identify plant disease characteristics of the multi-label identification model. Then, the MLP structure optimizes the model to prevent model degradation during the training. Meanwhile, the MLP further transforms the plant disease features to fit the model and enhance its feature representation ability^47^. Finally, this module generates context-rich tokens that contain multi-label information of plant diseases.

### Multi-label decoder module with residual structure

Studies have shown that with increasing accumulation, fusing information of different levels can improve the identification accuracy compared to other networks^50,51^. In this paper, a residual structure has been designed to connect compact spatial feature tokens containing low-level features and context-rich tokens containing high-level features. This avoids the loss of plant disease information after high-level extraction, which can enhance the ability to extract multi-label information of the model^52,53^. Now, the fused tokens are needed to project to multi-label results. To achieve this, we use the multi-label decoder module, which was first used in^54^. Adaptive feature embeddings are incorporated the fused tokens to guide the multi-label identification of plant diseases^51,55^.

The multi-label decoder module consists of two multi-label decoder blocks. Figure 3b illustrates that the multi-label decoder block with two inputs includes a multi-head self-attention, a multi-head cross-attention and an MLP. The first input is discriminative adaptive feature embeddings that are generated based on the multi-label identification task of plant diseases^56^. These embeddings are fed into the multi-head self-attention as the input Q, K and V. The outputs of the multi-head self-attention are then fed into the multi-head cross-attention as input Q. Studies have found that integrating feature information at different levels enhances the ability to extract information of the model^57^. Therefore, compact spatial feature tokens and context-rich tokens are fused, which are then fed into the cross-attention as input K and V. The correlations between the label and feature can be fully learned by cross-attention^48^. In the end, the outputs of the cross-attention are fed into the MLP to obtain the multi-label identification results of plant diseases.

## Experiments and results

### Dataset description

The dataset employed in this paper is the AI challenger 2018 dataset, which is sourced from the publicly available repositories and widely used in the research direction of plant disease identification^21^. It comprises 61 categories with a total of 31,718 plant disease images for training, 4540 for validation and 4514 for testing. This paper utilized 30,210 images from the training set and 4324 from the validation set, containing 56 categories after eliminating 5 categories that have only a few images. As the test set lacks of true labels, the training set adopts the method of proportional stratified sampling, which is further separated into two parts, i.e., the training and validation parts, in a ratio of 80% and 20%. The validation set is used for testing. So, the number of images for training, validation and testing are 24,159, 6054, and 4324, respectively. The 56 categories of plant diseases are displayed in Appendix A. Notably, there are 4 diseases that can infect different plants, such as scab which can infect both pepper and peach leaves. Assigning each type of disease to the same label can expand the number of labels, thereby preventing overfitting and enhancing the generalization performance of the model during training. Therefore, this study assigns multi-label to each image in the dataset. The multi-label is obtained in the form of one-hot embedding, which ultimately results in 34 categories of labels. For instance, if the single-label of an image is "Tomato Late Blight Serious", the multi-label of the image contains the three labels of "Tomato", "Late Blight", and "Serious", with the positions corresponding to these labels are encoded as 1 and other positions are encoded as 0. The dataset includes 12 categories of plants, and the information is displayed in Table 1. There are 19 categories of diseases, and the information is displayed in Table 2 and CZTD is the abbreviation of “Cerospora Zeaemavdis Tehon and Daniels”. In addition, the severity is divided into 3 categories, i.e., "Healthy", "General" and "Serious", and the information is displayed in Table 3.

**Table 1:** Plant statistics of the dataset used in this paper.

Name	Training set	Validation set	Test set
Apple	1382	348	247
Cherry	658	166	115
Citrus	3194	800	583
Corn	1824	454	325
Grape	2202	548	394
Peach	1500	378	268
Pepper	1353	336	241
Potato	1130	280	202
Pumpkin	1025	260	184
Soybean	1142	288	204
Strawberry	817	200	144
Tomato	7932	1996	1417

**Table 2:** Disease statistics of the dataset used in this paper.

Name	Training set	Validation set	Test set
Black Blight Fungus	551	140	99
Black Measles Fungus	738	184	133
Black Rot Fungus	675	168	120
Black Spot	287	76	52
Cedar Rust	146	36	26
CZTD	286	72	51
Early Blight	1126	280	201
Greening June	2903	724	531
Late Blight	1654	416	296
Leaf Blight	623	152	109
Leaf Mold	529	132	94
Leaf Spot	564	142	100
Powdery Mildew	1205	306	214
Puccinia Polysora	670	168	120
Scab	1831	460	326
Septoria Leaf Spot Fungus	980	248	175
Spider Mite Damage	649	164	116
Target Spot Bacteria	53	12	9
YLCV Virus	3107	780	555

**Table 3:** Severity statistics of the dataset used in this paper.

Name	training set	Validation set	Test set
General	7600	1893	1356
Serious	10,977	2767	1971
Healthy	5582	1394	997

**Figure 4:**
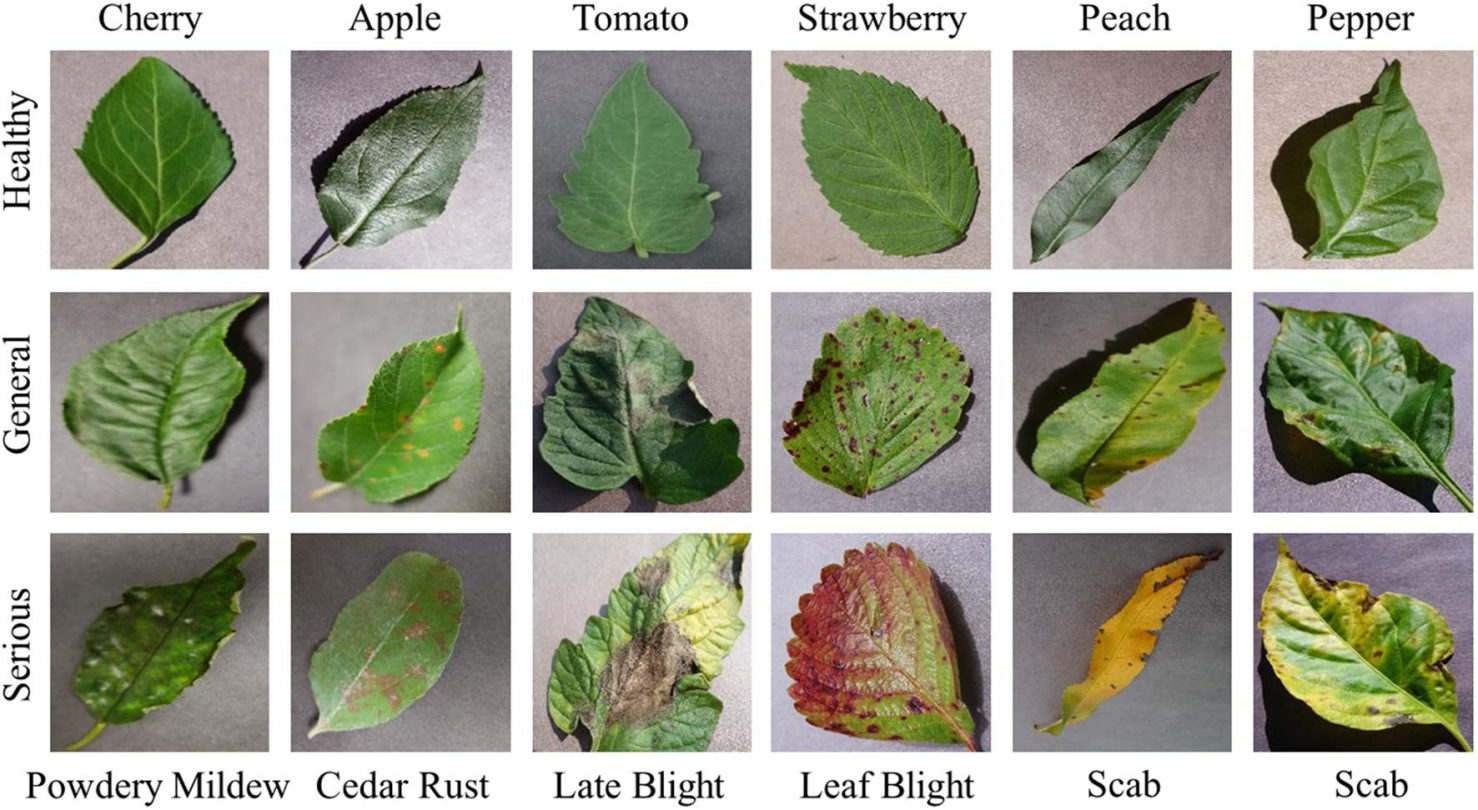
Illustration of the dataset used in the paper.

Figure 4 shows examples of dataset images. To further prevent overfitting during model training, data augmentation is implemented on the training set^58^. Specifically, the images are randomly cropped and resized to 224 × 224 pixels, and then randomly horizontally flipped. These two techniques effectively expand the dataset.

### Baseline models

**Figure 5:**
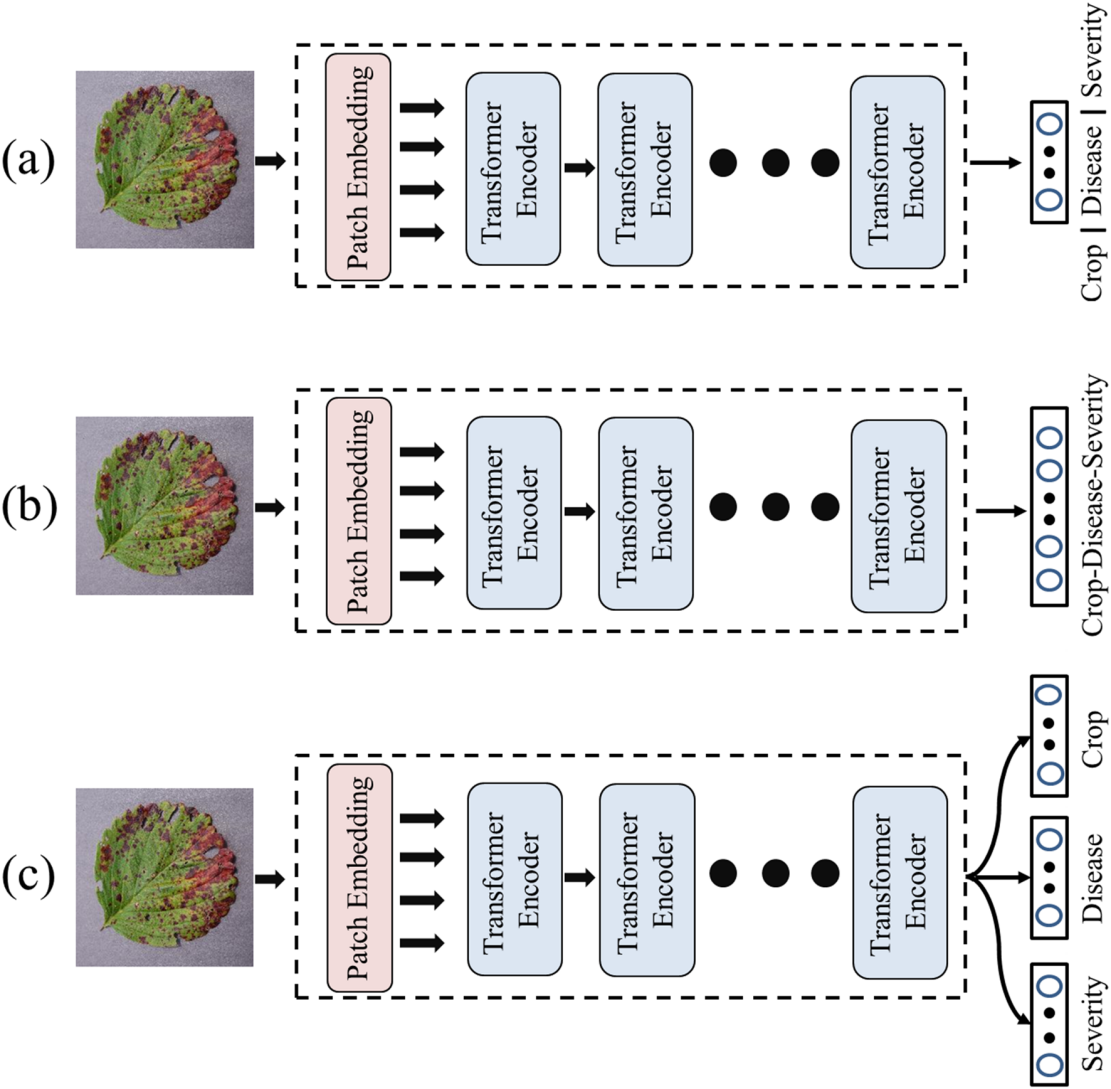
Illustration of the baseline models (**a**) Single nets, (**b**) Joint net, (**b**) Multi-task.

To evaluate the superior performance of the LDI-NET, it was compared to three types of baseline models shown in Fig. 5 that are widely employed to identify plant diseases. The first type is the single nets, which utilizes a single-branch model for several identifications^59^. The second type is the joint net, which identifies the plant-disease-severity combination with a single-branch model^60^. The last type is the multi-task model that shares a common backbone network and connects several branches to correspond to multiple tasks^24^.

In this paper, the single nets model uses the visual transformer^30^ as the backbone network for three tasks: the identification task of plant, disease, and severity, respectively. The joint net model uses the visual transformer to identify all 56 plant-disease-severity combinations. The multi-task model also uses a visual transformer as the backbone network and connects three fully connected layers to identify plant, disease and severity separately.

### Evaluation metrics

To evaluate the performance of the LDI-NET, four evaluation indexes are adopted, i.e., $\text{Precision}$, $\text{Recall}$, $\text{F}1\text{score}$ and $\text{Accuracy}$. $\text{Precision}$ is the ratio of true positive samples to the total number of samples predicted to be positive. $\text{Recall}$ is the ratio of true positive samples to the total number of positive samples. The $\text{F}1\text{score}$ , which combines $\text{Precision}$ and $\text{Recall}$ to yield a value between 0 and 1. A score of 1 represents the best model, while a score of 0 represents the worst model. $\text{Accuracy}$ is the ratio of correctly predicted images to the total number of predicted images. The expressions are as follows:

8 $$\text{Precision}=\frac{TP}{TP+FP}$$

9 $$\text{Recall}=\frac{TP}{TP+FN}$$

10 $$\text{F}1\text{score}=\frac{2*precision*recall}{precision+recall}$$

11 $$\text{Accuracy}=\frac{Number of correct predictions}{Total number of predictions}$$

where TP denotes True Positive, FP denotes False Positive and FN denotes False Negative^61^.

The $n$ categories of labels are imbalanced, so $\text{weighted} \text{Precision}$, $\text{weighted} \text{Recall}$ and $\text{weighted} \text{F}1\text{score}$ are used as the evaluation indexes. In order to avoid confusion, this paper uses $\text{Precision}$, $\text{Recall}$ and $\text{F}1\text{score}$ to represent $\text{weighted} \text{Precision}$, $\text{weighted Recall}$ and $\text{weighted} \text{F}1\text{score}$.

### Implementation details

The experimental hardware platform employed an Intel i9-12900K CPU and an NVIDIA 3090ti GPU. The experiments were conducted on the Ubuntu platform. The LDI-NET was implemented using the PyTorch framework. During the experiments, the LDI-NET was trained iteratively for 60 epochs, with a training batch size of 16. The adaptive moment estimation weight decay (AdamW) algorithm was adopted to optimize the network. To ensure faster convergence, StepLR method was implemented with an initial learning rate of 6.25e−6, a step size of 4 and a gamma of 0.5. To ensure reliability, all experiments were performed five times and the results were averaged.

### Experimental results

The Fig. 5 shows the three baseline models: single nets, join net and multi-task model. Note that the LDI-NET is a multi-label identification network, while the baseline models are single-label identification networks. Therefore, the LDI-NET identifies plant, leaf disease and severity simultaneously, while the baseline models identify them either combined or separately. The experimental results are presented in Table 4, which shows that the LDI-NET outperforms the baseline models. In terms of plant accuracy, severity accuracy and plant-disease-severity accuracy (PDSA), the LDI-NET achieves 99.42%, 88.55% and 87.40%, respectively. These values are at least 0.07%, 0.69%, and 0.88% higher than the other models, respectively. To further verify the significant difference between the results of LDI-NET and the three baseline models, we performed an independent samples t-test to analysis the five experimental results. Table 5 demonstrates that the t-test significances at Plant-Disease-Severity Accuracy of LDI-NET and the three baseline models are less than 0.001, indicating a significant difference between them.

**Table 4:** Accuracy of different networks for plant, disease, severity and plant-disease-severity (%).  Significant values are in bold.

Models	Single nets	Joint net	Multi-task	LDI-NET
Plant accuracy	99.28	99.35	99.28	**99.42**
Disease accuracy	**98.71**	98.44	98.38	98.68
Severity accuracy	87.86	87.47	87.51	**88.55**
Plant-disease-severity accuracy	86.52	86.26	85.82	**87.40**

**Table 5:** Independent sample t-test of LDI-NET and other baseline models. S represents Single nets, J represents Joint net, and M represents Multi-task.

		Levene's test for equality of variances		t-test for equality of means						
		F	Sig	t	*df*	Sig (2-tailed)	Mean difference	Std, error difference	95% confidence interval of the difference	
									Lower	Upper
S	Equal variances assumed	0.945	0.360	− 0.872	8.000	< 0.001	− 0.880	0.128	− 1.175	− 0.585
	Equal variances not assumed			− 0.872	6.695	< 0.001	− 0.880	0.128	− 1.185	− 0.574
J	Equal variances assumed	1.115	0.322	− 8.943	8.000	< 0.001	− 1.140	0.127	− 1.434	− 0.846
	Equal variances not assumed			− 8.943	6.629	< 0.001	− 1.140	0.127	− 1.445	− 0.835
M	Equal variances assumed	0.129	0.728	− 9.436	8.000	< 0.001	− 1.580	0.167	− 1.966	− 1.194
	Equal variances not assumed			− 9.436	7.808	< 0.001	− 1.580	0.167	− 1.966	− 1.192

The above results demonstrate that the LDI-NET, based on a multi-label method, has a more powerful ability to extract features, resulting in more accurate identification of plant diseases.

For a more detailed analysis of the experimental results, the identification accuracy for each plant, disease and severity is presented in Tables 6, 7 and 8, respectively. The results demonstrate that the LDI-NET achieves over 97% for each of the twelve plants and eight plants are superior or equal. For the nineteen diseases, the LDI-NET shows a superiority or parity for fourteen diseases, with six diseases achieving 100% and five diseases achieving over 99%. For the three severities, the LDI-NET outperforms the other three models in identifying “Healthy” and “General”, particularly with the latter being at least 1.7% higher than the other models. Identifying “Healthy” is generally straightforward due to their distinctive features, but distinguishing between “General” and “Serious” can be challenging, resulting in low accuracy rates for severity identification as shown in Table 4. The LDI-NET greatly surpasses the three baseline models in the identification of “General”. In all, the LDI-NET achieves better performance than the single nets, join net and multi-task.

**Table 6:** Accuracy of different networks for different plants (%).

Models	Single nets	Joint net	Multi-task	LDI-NET
Apple	97.17	96.76	96.76	97.57
Cherry	100	100	100	100
Citrus	100	100	100	100
Corn	99.69	100	100	100
Grape	100	100	100	100
Peach	97.39	97.76	97.39	97.76
Pepper	99.59	99.59	100	99.59
Potato	97.52	96.53	97.03	99.01
Pumpkin	100	100	100	100
Soybean	100	100	99.51	99.51
Strawberry	100	100	100	99.31
Tomato	99.17	99.58	99.36	99.44

**Table 7:** Accuracy of different networks for different diseases (%).

Models	Single nets	Joint net	Multi-task	LDI-NET
Black Blight Fungus	100	100	100	100
Black Measles Fungus	100	99.25	99.25	100
Black Rot Fungus	99.17	99.17	99.17	99.17
Black Spot	92.31	90.38	92.31	92.31
Cedar Rust	100	100	100	100
CZTD	90.20	80.39	86.27	86.27
Early Blight	98.00	96.52	96.02	96.52
Greening June	100	100	100	100
Late Blight	98.31	99.32	97.64	97.97
Leaf Blight	100	100	100	99.08
Leaf Mold	97.87	95.74	96.81	97.87
Leaf Spot	92.00	96.00	94.00	96.00
Powdery Mildew	99.53	100	100	100
Puccinia Polysora	100	100	99.25	100
Scab	99.39	98.77	98.77	99.39
Septoria Leaf Spot Fungus	97.71	97.71	97.14	99.43
Spider Mite Damage	95.69	95.69	95.69	95.69
Target Spot Bacteria	100	88.89	77.78	77.78
YLCV Virus	99.64	99.28	98.92	99.64

**Table 8:** Accuracy of different networks for different severities (%).

Models	Single nets	Joint net	Multi-task	LDI-NET
Healthy	98.40	99.40	98.80	**99.50**
General	78.91	77.80	79.79	**81.49**
Serious	**88.19**	88.08	87.16	87.87

Significant values are in bold.

### Analysis of identification results

**Figure 6:**
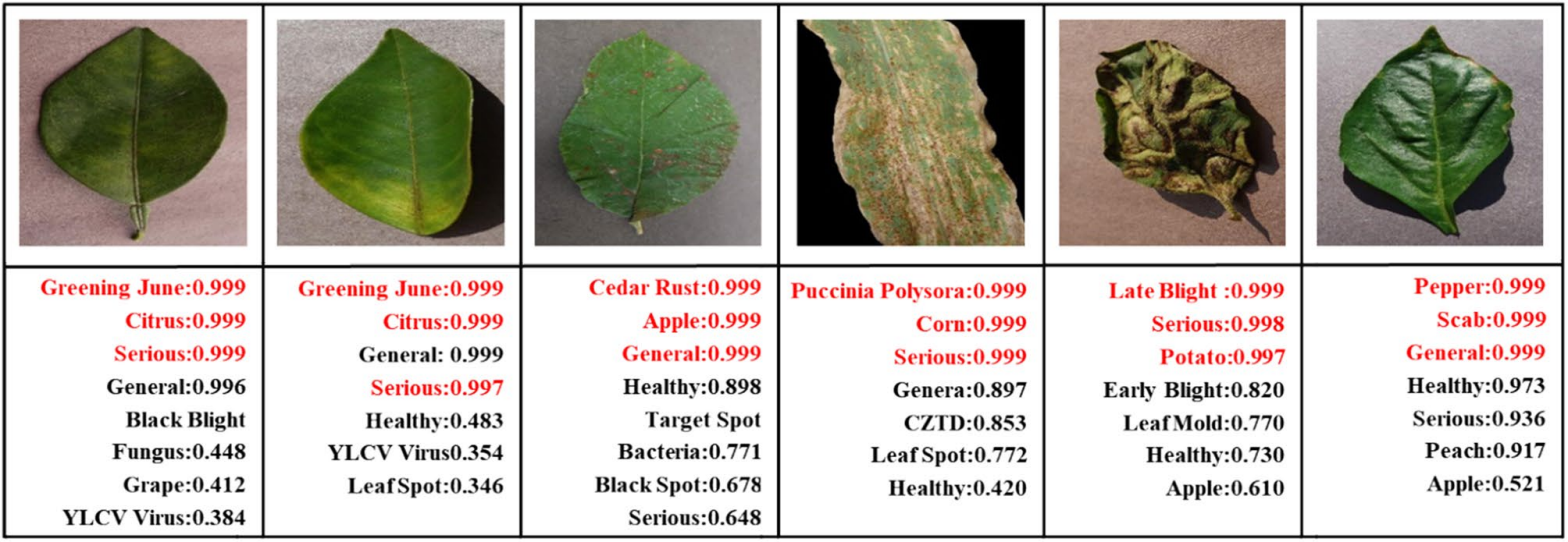
The illustration of multi-label identification results.

Figure 6 shows the identification results of the LDI-NET. The top seven predicted labels and their corresponding scores for each test image are visually depicted. Correct labels are denoted in red and incorrect labels in black, making them easily distinguishable. It is noticeable that the most predicted scores for correct labels are significantly higher than those for incorrect labels, which demonstrates the remarkable identification ability of LDI-NET to identify different labels of plant and disease. For instance, in the first image, “Greening June” is predicted with a score of 0.999, while “YLCV Virus” is only predicted with a score of 0.384. Similarly, in the last image, “Pepper” is predicted with a score of 0.999, while “Apple” is only predicted with a score of 0.521. These findings reveal that distinguishing between “Greening June” and “YLCV Virus”, as well as “Pepper” and “Apple”, is relatively effortless due to their distinct features. However, for severity, the distinction is less clear. As shown in the example of the second image, where the predicted score for “Serious” is 0.997 and the score for “General” is 0.999. The prediction is incorrect as it implies that there are no distinct features to differentiate severity, leading to some confusion between the “General” and “Serious”. This is reflected in the lower accuracy of the “General” and “Serious” compared to the “Healthy”, as shown in Table 8. These results demonstrate that the LDI-NET can achieve the multi-label identification and distinguish between features and labels of plant diseases based on predicted scores.

## Discussion

### Visualization of deep feature

Visualizing the feature layers can assist in exploring the learning process of the LDI-NET for identifying plant diseases, thereby improving the interpretability of the model. In this paper, the GradCAM technique^62^ is used to visualize the regions of interest for plant disease images. In the results of the heat maps, deeper shades of red indicate that the attention of the model is more focused on that area, while yellow indicates less attention, and blue indicates the least attention^14^.

**Figure 7:**
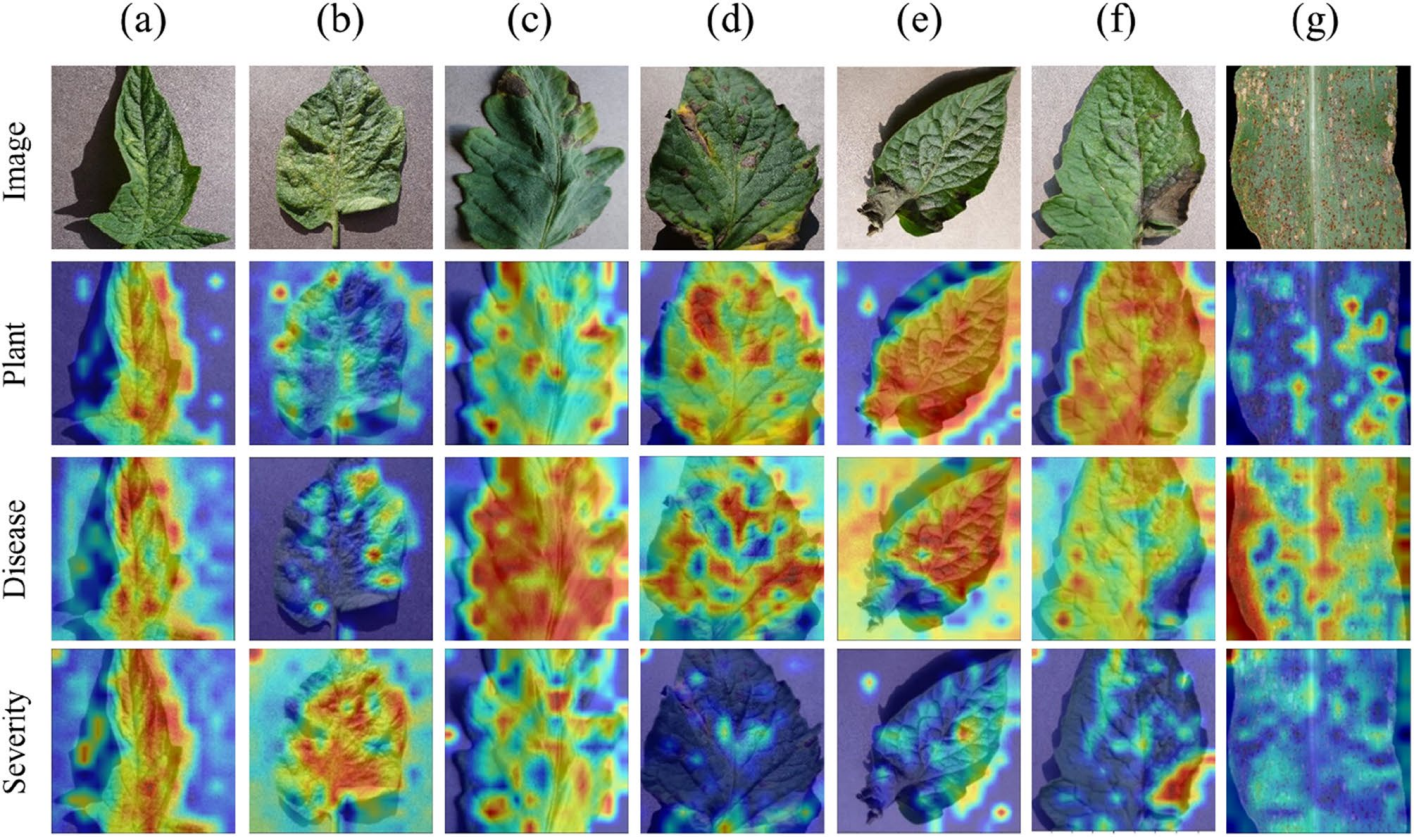
Visualization of deep feature (**a**) Tomato spider mite damage general, (**b**) Tomato spider mite damage serious, (**c**) Tomato early blight general, (**d**) Tomato early blight serious, (**e**) Tomato late blight general, (**f**) Tomato late blight serious, (**g**) Corn puccinia polysora serious.

Figure 7 illustrates that the LDI-NET focuses on the leaf region when identifying plant type, leaf disease, or severity. Attention is usually focused on the entire leaf for the plants, while for leaves with distinct characteristics, such as corn, more attention is focused on the interior of the leaf. For the diseases and severities, attention is focused on the diseased areas. This improves the ability to identify diseases and severities. By observing the visualization results, it is found that the LDI-NET could identify different regions of the leaves for different types of labels. It improves the efficiency of identifying plant diseases.

### Selection of feature extractor

To explore the effectiveness of the feature tokenizer module, this study employed 6 prevalent deep learning models categorized into three groups: VGG11 and VGG16, ResNet18 and ResNet50 and swin transformer tiny and swin transformer base, alongside the proposed feature tokenizer module (patch size = 16) and feature tokenizer module (patch size = 32) which combine the CNN and transformer. These backbone networks are utilized to extract feature. In terms of evaluation indexes, $\text{Precision}$, $\text{Recall}$, $\text{F}1\text{score}$ and $\text{PDSA}$ were adopted.

According to the experimental results presented in Table 9, the feature tokenizer module (FTM) with a patch size (ps) of 16 is the most effective feature extraction network. It outperforms the feature tokenizer module with a ps of 32 in terms of $\text{Precision}$, $\text{Recall}$, $\text{F}1\text{score}$ and $\text{PDSA}$, achieving 95.28%, 95.29%, 95.28%, and 87.40%, respectively. Comparing VGG11 and VGG16, ResNet18 and ResNet50, swin transformer tiny (SwinT) and swin transformer base (SwinB), as well as the feature tokenizer module (ps = 32) and feature tokenizer module (ps = 16), it can be concluded that deeper feature extraction networks with larger receptive fields are better at capturing useful multi-label information. This results in stronger feature extraction abilities and better final identification results for plant diseases.

**Table 9:** Recognition performance with different backbone networks presented as $\text{Precision}$, $\text{Recall}$, $\text{F}1\text{score}$ and PDSA (%).

Backbone	VGG11	VGG16	ResNet 18	ResNet 50	SwinT	SwinB	FTM (ps = 32)	FTM (ps = 16)
$\text{Precision}$	93.72	94.12	93.43	94.49	94.78	94.92	95.00	**95.28**
$\text{Recall}$	93.65	94.08	93.34	94.46	94.65	94.91	94.99	**95.29**
$\text{F}1\text{score}$	93.64	94.08	93.35	94.46	94.67	94.91	94.99	**95.28**
PDSA	83.21	84.46	82.74	85.50	85.68	86.52	86.68	**87.40**

Significant values are in bold.

Based on the results above, it is demonstrated that the feature tokenizer module is the most suitable backbone network than others. This is because it utilizes both CNN and transformer to capture local and long-range global information, allowing the model to transform image data into highly tokenized sequences and extract fine-grained features more effectively. Therefore, the feature tokenizer module is the optimal choice among the tested backbone networks.

### Rationality verification of the number of encoder and decoder blocks

To explore the effect of the number of token encoder and multi-label decoder blocks on the performance of LDI-NET, this study conducted ablation experiments to determine the optimal combination. Nine combinations were designed and tested. The results of the ablation experiment are illustrated in Table 10, which reveal that varying the number of token encoder blocks and multi-label decoder blocks has an impact on the performance of the LDI-NET. Specifically, when the number of token encoder blocks is held constant, increasing the number of multi-label decoder blocks from 1 to 3 initially improves the identification performance, but this improvement is followed by a decline. Similarly, fixing the number of multi-label decoder blocks and increasing the number of token encoder blocks from 0 to 2 initially improves the performance, which then stabilizes. Notably, when both the number of token encoder blocks and multi-label decoder blocks are set to 2, the model achieves the best results, with 95.28%, 95.29%, 95.28%, and 87.40% for $\text{Precision}$, $\text{Recall}$, $\text{F}1\text{score}$ and $\text{PDSA}$, respectively, outperforming the other combinations. Increasing the number of token encoder blocks and multi-label decoder blocks beyond this point may enhance the robustness but weaken the overall functionality of the model^63^. Therefore, choosing the appropriate number of token encoder blocks and multi-label decoder blocks requires balancing robustness and overall performance. In this paper, the optimal number of token encoder blocks and multi-label decoder blocks is both 2.

**Table 10:** Identification performance with different number of token encoder and multi-label decoder blocks presented as $\text{Precision}$, $\text{Recall}$, $\text{F}1\text{score and}$ PDSA (%).

	Num of encoder			Num of decoder			$\text{Precision}$	$\text{Recall}$	$\text{F}1\text{score}$	$\text{PDSA}$
	0	1	2	1	2	3				
1	√			√			95.03	95.04	95.03	86.70
2	√				√		95.27	**95.29**	95.27	87.37
3	√					√	95.16	95.18	95.16	87.07
4		√		√			95.20	95.22	95.20	87.19
5		√			√		95.26	95.27	95.25	87.39
6		√				√	94.93	94.93	94.93	86.36
7			√	√			95.11	95.13	95.11	86.96
8			√		√		**95.28**	**95.29**	**95.28**	**87.40**
9			√			√	94.94	94.96	94.93	86.53

Significant values are in bold.

## Conclusion

In this paper, we propose a novel LDI-NET for identifying plant diseases using a multi-label method. In order to improve the performance of LDI-NET, we construct the feature tokenizer module as the backbone network, leveraging the strengths of CNN and transformer. This module can efficiently extract local and long-range global features and obtain compact feature tokens. To establish relationships among different features, the token encoder module is utilized to facilitate the exchange of multi-label information between tokens. Additionally, the multi-label decoder module with a residual structure is utilized to output multi-label identification results simultaneously by integrating fused tokens with adaptive feature embeddings. The LDI-NET outperforms the other three baseline models (i.e., single nets, joint net, and multi-task) in the AI challenger 2018 dataset. In terms of plant accuracy, severity accuracy and plant-disease-severity accuracy, the LDI-NET achieves accuracies with 99.42%, 88.55%, and 87.40%, respectively. These results are at least 0.07%, 0.69%, and 0.88% higher than the other baseline models. One significant contribution of this paper is the use of the multi-label method for identifying plant diseases, which combines the advantages of single-branch and multi-branch methods. Additionally, the feature tokenizer module captures information, the token encoder module exchanges information, and the multi-label decoder module correlates feature and label, all of which improve the identification performance of LDI-NET. Our goal is to improve the accuracy of our research and develop a more lightweight and practical model for deployment. We hope our research can have an effect on agricultural disease prevention.

### Supplementary Information

Supplementary Table 1.

## Data Availability

The datasets generated during and/or analysed during the current study are available from the corresponding author on reasonable request.
