# Supplementary material for: A novel plant type, leaf disease and severity identification framework using CNN and transformer with multi-label method
Source: Sci Rep. 2024 May 22;14:11664. doi: 10.1038/s41598-024-62452-x (PMC11111674; doi:10.1038/s41598-024-62452-x)
Supplement: Supplementary file 1 — Supplementary Table 1. [file 41598_2024_62452_MOESM1_ESM.docx]

| **Plant** | **Disease** | **Severity** | **Training Set** | **Validation Set** | **Test Set** |
| --- | --- | --- | --- | --- | --- |
| Apple | [Black Spot](http://www.baidu.com/link?url=Mu8UOH_MMx1VjBGTy7dtaf-r878tasoMiN6fV1v6T-EbbpRjfhfNj8v7j5Yssqn3yKEb1gthzhMSG5cPpMf4G_X3-zdRkA10ZyF8Um07Rji) | General | 167 | 44 | 30 |
|  |  | Serious | 120 | 32 | 22 |
|  | Cedar Rust | General | 114 | 28 | 20 |
|  |  | Serious | 32 | 8 | 6 |
|  |  | Healthy | 949 | 236 | 169 |
| Corn | CZTD | General | 152 | 39 | 27 |
|  |  | Serious | 134 | 33 | 24 |
|  | Leaf Spot | General | 166 | 42 | 29 |
|  |  | Serious | 398 | 100 | 71 |
|  | Puccinia Polysor | General | 387 | 96 | 69 |
|  |  | Serious | 283 | 72 | 51 |
|  |  | Healthy | 304 | 72 | 54 |
| Cherry | Powdery Mildew | General | 92 | 24 | 12 |
|  |  | Serious | 88 | 22 | 18 |
|  |  | Healthy | 478 | 120 | 85 |
| Citrus | Greening June | General | 1464 | 364 | 269 |
|  |  | Serious | 1439 | 360 | 262 |
|  |  | Healthy | 291 | 76 | 52 |
| Grape | Black Blight Fungus | General | 49 | 12 | 9 |
|  |  | Serious | 502 | 128 | 90 |
|  | Black Measles Fungus | General | 403 | 100 | 74 |
|  |  | Serious | 335 | 84 | 59 |
|  | Black Rot Fungus | General | 305 | 76 | 54 |
|  |  | Serious | 370 | 92 | 66 |
|  |  | Healthy | 238 | 56 | 42 |
| Peach | Scab | General | 685 | 172 | 122 |
|  |  | Serious | 614 | 156 | 110 |
|  |  | Healthy | 201 | 50 | 36 |
| Pepper | Scab | General | 231 | 56 | 40 |
|  |  | Serious | 301 | 76 | 54 |
|  |  | Healthy | 821 | 204 | 147 |
| Potato | Early Blight | General | 163 | 40 | 29 |
|  |  | Serious | 410 | 100 | 73 |
|  | Late Blight | General | 203 | 48 | 36 |
|  |  | Serious | 354 | 92 | 64 |
| Pumpkin | Powdery Mildew | General | 255 | 64 | 46 |
|  |  | Serious | 770 | 196 | 138 |
| Soybean |  | Healthy | 1142 | 288 | 204 |
| Strawberry | Leaf Blight | General | 156 | 36 | 27 |
|  |  | Serious | 467 | 126 | 82 |
|  |  | Healthy | 194 | 48 | 35 |
| Tomato | Early Blight | General | 199 | 52 | 36 |
|  |  | Serious | 354 | 88 | 63 |
|  | Late Bligh | General | 212 | 52 | 38 |
|  |  | Serious | 885 | 224 | 158 |
|  | Leaf Mold | General | 261 | 64 | 46 |
|  |  | Serious | 268 | 68 | 48 |
|  | Septoria Leaf Spot Fungus | General | 337 | 84 | 60 |
|  |  | Serious | 643 | 164 | 115 |
|  | Spider Mite Damage | General | 434 | 108 | 77 |
|  |  | Serious | 215 | 56 | 39 |
|  | Target Spot Bacteria | General | 35 | 8 | 4 |
|  |  | Serious | 18 | 4 | 5 |
|  | VLCY Virus | General | 1130 | 284 | 202 |
|  |  | Serious | 1977 | 496 | 353 |
|  |  | Healthy | 964 | 244 | 173 |
